# Preparation of Dual-Layered Core–Shell Fe_3_O_4_@SiO_2_ Nanoparticles and Their Properties of Plasmid DNA Purification

**DOI:** 10.3390/nano11123422

**Published:** 2021-12-17

**Authors:** Jin Soon Han, Gye Seok An

**Affiliations:** 1 Division of Materials Science and Engineering, Hanyang University, Seoul 04763, Korea; hanjsoon@hanyang.ac.kr; 2 Department of Advanced Material Engineering, Kyonggi University, Suwon 16227, Korea

**Keywords:** Fe_3_O_4_, nanocomposites, magnetic properties, SiO_2_, plasmid DNA purification

## Abstract

The rapid purification of biomaterials such as DNA, RNA, and antibodies has attracted extensive attention, and research interest has increased further with the COVID-19 pandemic. In particular, core–shell-structured superparamagnetic nanoparticles have been continuously studied for their application as biopurification materials. It has been reported that Fe_3_O_4_@SiO_2_ nanoparticles are one of the most promising candidates for separating nucleic acids via a simple and rapid process. This study proposed a fabrication method for dual-layered Fe_3_O_4_@SiO_2_ nanoparticles, in which the density of the SiO_2_ shell was controlled using an intermediate surfactant during the SiO_2_ coating. After the fabrication of dual-layered Fe_3_O_4_@SiO_2_ nanoparticles, structural, morphological, and magnetic analyses were conducted. The results showed that the Fe_3_O_4_ nanoparticles were surrounded by a dense layer 15.6~27.9 nm thick and a porous layer 24.2~44.4 nm thick, and had superparamagnetic properties with high saturated magnetization at room temperature (86.9 emu/g). Then, the optimal conditions for the biopurification material were suggested based on analysis of the selective separation of plasmid DNA.

## 1. Introduction

Superparamagnetic nanoparticles are considered promising materials in various fields owing to their magnetic properties [1,2,3,4,5,6,7]. Biomedical engineering is one of the most promising fields, and it requires rapid processes for the separation and purification of biomaterials. Rapid RNA purification has become an urgent problem in the biomedical field, especially because of the COVID-19 pandemic. Therefore, superparamagnetic nanoparticles have been utilized for separating biomaterials as an advanced substitute for non-magnetic nanoparticles or membranes [8,9,10,11]. To purify biomaterials using superparamagnetic nanoparticles, a surface functionalization process is necessary, and many related studies have been reported based on both basic research and practical applications [6,8,9].

Since the core–shell structure could take advantage of both an Fe_3_O_4_ core, which could control nanoparticles through its superparamagnetic properties, and an SiO_2_ shell, which can be used for purifying biomaterials such as nucleic acid and antibodies, surface functionalization through silane grafting, and enhancing chemical and thermal stabilities, core–shell-structured Fe_3_O_4_@SiO_2_ nanoparticles have received extensive attention, and a large number of studies have been conducted on the fabrication method and application of Fe_3_O_4_@SiO_2_ nanoparticles [12,13,14,15,16]. Most fabrication studies of Fe_3_O_4_@SiO_2_ nanoparticles have been conducted using the sol–gel method with silane precursors such as tetraethyl orthosilicate (TEOS) [6,13] and tetramethyl orthosilicate (TMOS) [2,3]. It has been reported that biomaterials, such as plasmid DNA, RNA, and antibodies, have been successfully purified using Fe_3_O_4_@SiO_2_ nanoparticles in high-speed separation processes [17,18]. Additionally, the SiO_2_ shell acts as a protective layer, which protects the Fe_3_O_4_ core (a relatively unstable structure) against chemical and heat effects. After initial research on the separation of biomaterials using Fe_3_O_4_@SiO_2_ nanoparticles, studies have been focusing on applications based on their DNA-separating properties [9,17,19,20,21,22].

However, the demand for a higher efficiency of DNA purification is consistent, and studies have been carried out in order to enhance the concentration of separated DNA. [8,12,23]. Owing to the above-mentioned advantages provided by the SiO_2_ shell, many studies have been conducted to maximize DNA purification efficiency by extending the surface area of the SiO_2_ shell [3,8,12,23]. Most studies on the fabrication of complex Fe_3_O_4_@SiO_2_ nanoparticles have reported mesoporous or hollow SiO_2_ shell structures with nanosized pores [8,12,23]. Studies have also reported successful DNA purification and have shown an increase in the amount of purified DNA on the application of SiO_2_ shells with extended surface areas [12]. However, among the increased surface area there must be some areas on which DNA could not be attached, and the efficiency of an increased surface area through porous SiO_2_ shell structures has not been presented yet. Additionally, because the nanoscale pores could potentially decrease the stability of Fe_3_O_4_@SiO_2_ nanoparticles by forming a channel in the SiO_2_ shell [12], the different structures of the SiO_2_ shell have to be presented.

In this study, we proposed a preparation method based on the core–shell structure for dual-layered Fe_3_O_4_@SiO_2_. We validated the performance of plasmid DNA purification based on the structuring of nanoparticles on the SiO_2_ shell surface. Various structures of the SiO_2_ layer, including normal, porous, and dual-layered structures, were compared based on their surface characteristics to determine the relationship between surface characteristics and DNA purification efficiencies. In addition, we discuss the effects of the types of shell structures on the dispersion and magnetic properties of Fe_3_O_4_@SiO_2_ nanoparticles, which could further affect the plasmid DNA purification.

## 2. Materials and Methods

### 2.1. Materials

Ferric chloride hexahydrate (FeCl_3_.6H_2_O, >97%, Sigma-Aldrich, Burlington, MA, USA), sodium acetate (NaOAc, 99.995%, Sigma-Aldrich, Burlington, MA, USA), and ethylene glycol (EG, >99.5% Samchun Chemical, Seoul, Korea) were used for Fe_3_O_4_ synthesis. To build a coating layer on the surface of the Fe_3_O_4_, TEOS (98%, Sigma-Aldrich, Burlington, MA, USA) was prepared as a silica precursor, and hexadecyltrimethylammonium bromide (CTAB, >98%, Sigma-Aldrich, Burlington, MA, USA) was used as a surfactant. Additionally, ammonia solution (NH_4_OH, 28–30 wt% stock solution in water, Junsei, Tokyo, Japan) and ethyl alcohol were utilized for the sol−gel reaction of the silica precursor. To purify the plasmid DNA (Axygen, Union City, CA, USA), binding buffer (5 M Gu-HCl, 20 mM Tris-HCl, pH 6.6), washing buffer (10 mM Tris-HCl, pH 7.5; 80% ethanol buffer, pH 6.5), and elution buffer (10 mM Tris-HCl, pH 8.0) were prepared without further purification.

### 2.2. Synthesis of Fe_3_O_4_ Nanoparticles via Polyol Method

Synthesis of the Fe_3_O_4_ nanoparticles was carried out based on a previously reported polyol method [24], in which 0.01 mol of FeCl_3_∙6H_2_O was dissolved in 0.3 mL of distilled water, and 0.05 mol of NaOAc was dissolved in 0.7 mol of EG. Subsequently, these solutions were mechanically mixed at 130 RPM in a 3 L round-bottom flask. The mixture was heated at 70 °C for 24 h in order to induce the first-phase transformation of the iron precursor. Then, the mixture was heated to boiling point under reflux until the yellow color of the solution turned to reddish brown and for the last the color of the whole solution changed into black. This dark-colored solution was cooled naturally, and the synthesized nanoparticles were separated using a magnet on the outer wall. The separated nanoparticles were washed with ethanol and distilled water several times before use.

### 2.3. Fabrication of Core–Shell Fe_3_O_4_@SiO_2_ (Dense) Nanoparticles

The prepared Fe_3_O_4_ nanoparticles (1 g) were dispersed in 500 mL of distilled water and sonicated for 30 min. Additionally, 200 mL of ammonia solution was prepared as 5 wt% of the composition, and the magnetically collected Fe_3_O_4_ nanoparticles were dispersed in the 200 mL of ammonia solution at room temperature. This solution was heated at 80 °C for 3 h with mechanical stirring at 300 RPM for surface treatment prior to SiO_2_ fabrication. After the surface treatment had finished, the solution was naturally cooled to room temperature, and 3 mL of TEOS diluted in 20 mL of ethanol was prepared in the syringe. With the syringe pump, the TEOS solution was slowly injected into the mixture with a feeding rate of 0.08 mL/min and consistent mechanical stirring. This reaction was carried out for 15 h and the solution was magnetically collected and washed several times with ethanol and distilled water, with vigorous sonication for 10 min after the reaction was completed.

### 2.4. Fabrication of Porous and Dual Core–Shell Fe_3_O_4_@SiO_2_ Nanoparticles

The surface treatment with ammonia solution mentioned in Section 2.3 was used on the as-prepared Fe_3_O_4_ nanoparticles prior to this stage. An amount of 1 g of surface-treated Fe_3_O_4_ nanoparticles or dense Fe_3_O_4_@SiO_2_ nanoparticles was dispersed in 80 mL of ethanol, and 1 wt% of CTAB solution was prepared with 100 mL of distilled water. These solutions were mixed to form a suspension in a 500 mL round-bottom flask with 300 RPM of mechanical stirring, and heated for 3 h at 80 °C. Then, the mixture was naturally cooled to room temperature. In total, 3 mL of TEOS diluted with 20 mL of ethanol was slowly injected into the mixture with a feeding rate of 0.08 mL/min via syringe pump. This reaction was carried out for 15 h and the solution was magnetically collected and washed several times with ethanol and distilled water, with vigorous sonication for 10 min after the reaction was completed.

### 2.5. Plasmid DNA Purification Using Fe_3_O_4_@SiO_2_ Nanoparticles

To separate the plasmid DNA from the Fe_3_O_4_@SiO_2_ nanoparticles, 30 μg of the plasmid DNA was mixed with the magnetic nanoparticles (5 mg/mL). A binding buffer was used as the solvent, and the solution was gently mixed for 5 min. Subsequently, the magnetic nanoparticles were magnetically separated and washed with 600 μL of washing buffer by gentle shaking. The attached plasmid DNA was desorbed from the magnetic nanoparticles using 100 μL of an elution buffer, and the supernatant was centrifuged with DNase-/RNase-free tubes after the nanoparticles were magnetically separated.

### 2.6. Characterization

The surface functional groups of the prepared Fe_3_O_4_ and Fe_3_O_4_@SiO_2_ nanoparticles were determined using Fourier-transform infrared spectroscopy (FT-IR, IRAffinity-1 S, Shimadzu, Japan) with at least 15 times scan and 2 cm^−1^ resolution. The structures of the Fe_3_O_4_ and Fe_3_O_4_@SiO_2_ nanoparticles were measured using X-ray diffraction (XRD, UltimaIV, Rigaku, Tokyo, Japan) with CuKα radiation (λ = 1.54178 Å) at a 2θ range of 20–80°. The sizes and morphologies of the Fe_3_O_4_ nanoparticles and SiO_2_ coating layer were observed using high-resolution transmission electron microscopy (HR-TEM, Tecnai G2 F30 S-Twin, FEI, Eindhoven, Netherlands). To measure the surface area, we conducted a surface area analysis based on the Brunauer−Emmett−Teller method (BET, ASAP 2010 M, Micromeritics, Norcross, GA, USA) with nitrogen gas under 77 K for the range P/P_0_ = 0.05–0.3. The dispersion properties of Fe3O4 and Fe3O4@SiO2 nanoparticles were characterized using dynamic light scattering (DLS; Zetasizer Nano, Malvern Instruments, Malvern, UK). The magnetic hysteresis loops of the Fe3O4 and Fe3O4@SiO2 nanoparticles were determined using a vibrating sample magnetometer (VSM, #73002 VSM system, Lake Shore Cryotronics Inc., Westerville, OH, USA) to evaluate their magnetic characteristics. Absorbance monitoring (μQuant™ Microplate Spectrometer, BioTek Instruments Inc., VT, USA) with agarose gel electrophoresis using a 3000-Xi power supply (Bio-Rad, Hercules, CA, USA) was used to analyze the DNA purification ability.

## 3. Results

**Figure 1:**
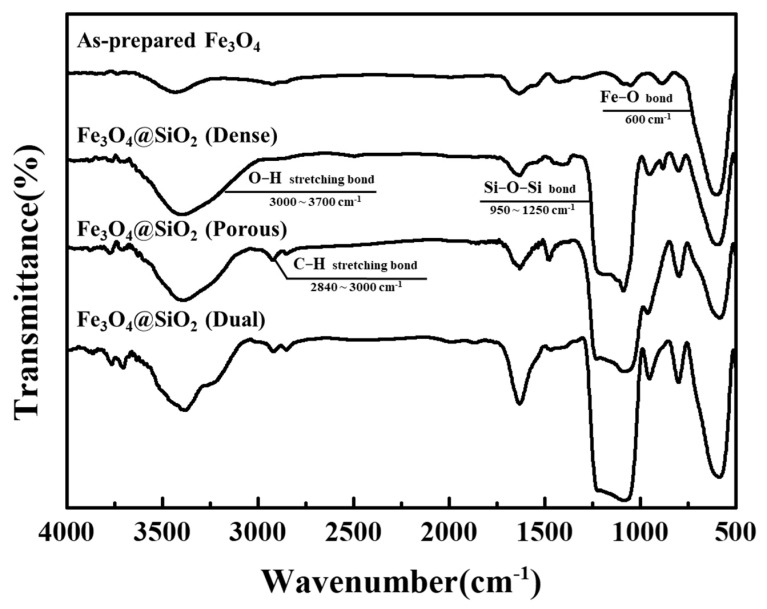
FT-IR spectra of as-prepared Fe_3_O_4_ and Fe_3_O_4_@SiO_2_ nanoparticles.

The FT-IR spectra of the prepared Fe_3_O_4_ and Fe_3_O_4_@SiO_2_ nanoparticles are shown in Figure 1 and the peak assignment between 500 and 2000nm are shown in Table 1. In all the spectra, a specific bond was observed at 600 cm^−^^1^, which corresponded to the Fe–O bonding. In the case of the dense Fe_3_O_4_@SiO_2_ complex, an intense peak around 1250–950 cm^−^^1^ and a broad peak at 3700–3000 cm^−^^1^ were observed in the FT-IR spectrum, which corresponded to the Si–O–Si bonds and O–H stretching bonds [6,13,25]. In the spectra of the porous and dual Fe_3_O_4_@SiO_2_, the Si–O–Si bond peak was maintained, but two peaks at wavelength ranges of 3000–2840 cm^−^^1^ (attributed to the C–H stretching bond) and 1700–1500 cm^−^^1^ replaced the corresponding peak of the O–H stretching bond [12,13]. The formation of these new bonds indicates that the CTAB affected the coating layer or remained intact after coating.

**Figure 2:**
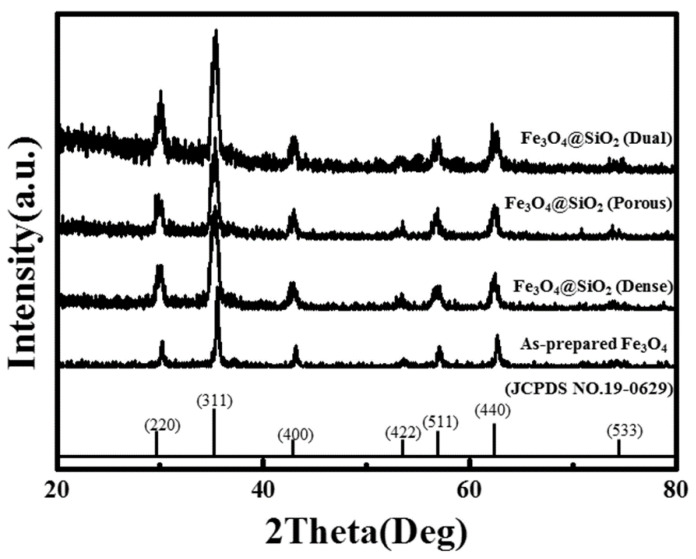
X-ray diffraction patterns of as-prepared Fe_3_O_4_ and Fe_3_O_4_@SiO_2_ nanoparticles.

XRD patterns of the prepared Fe_3_O_4_ nanoparticles and Fe_3_O_4_@SiO_2_ nanoparticles are shown in Figure 2. The diffraction pattern of the prepared Fe_3_O_4_ showed significantly distinguished peaks at 35.4°, 30.1°, and 62.5°, which indicate the (311), (220), and (440) planes of the inverse spinel crystal structure, respectively. This diffraction pattern was almost the same as that of the JCPDS card for Fe_3_O_4_ nanoparticles. (JCPDS No. 19-0629) [26]. In the case of the dense Fe_3_O_4_@SiO_2_, noise was observed under the 40° area and the width of each peak increased, especially at lower angles. This occurred because the surface of the Fe_3_O_4_ nanoparticles was under stress between the Fe_3_O_4_ core and the SiO_2_ shell, and the inter-planar distance was slightly increased [14,27]. Additionally, this phenomenon occurred in the porous Fe_3_O_4_@SiO_2_ and dual Fe_3_O_4_@SiO_2_. The noise in the 40° area was stronger in these cases than that of the dense Fe_3_O_4_@SiO_2_. This noise indicates the possibility of an amorphous phase on the surface. The FT-IR spectra indicate the formation of an amorphous phase with Si–O–Si bonding on the surface of the Fe_3_O_4_ nanoparticles as an SiO_2_ coating layer [14].

**Figure 3:**
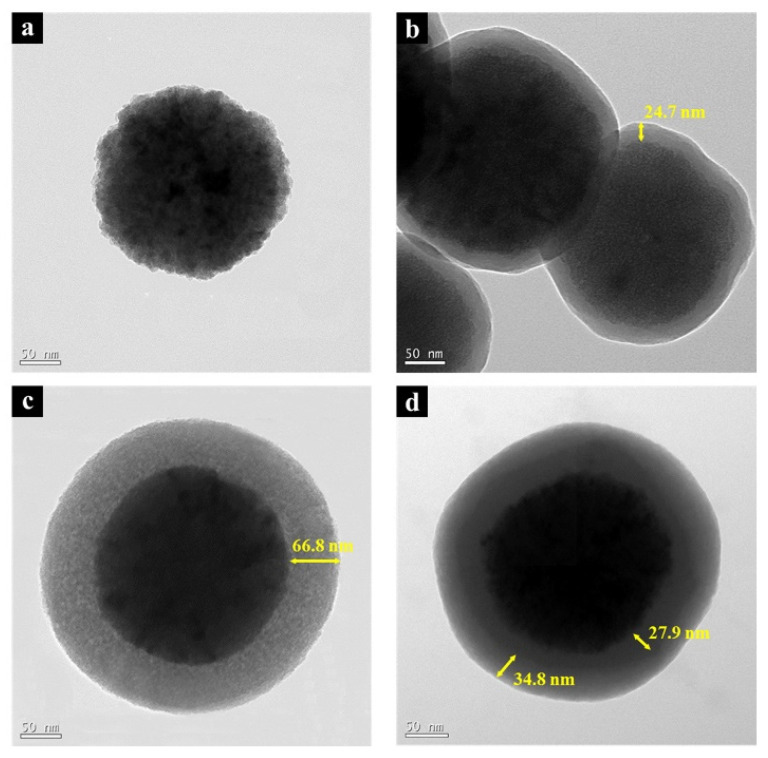
(**a**) TEM images of as-prepared Fe_3_O_4_, (**b**) Fe_3_O_4_@SiO_2_ (dense), (**c**) Fe_3_O_4_@SiO_2_ (porous), and (**d**) Fe_3_O_4_@SiO_2_ (dual).

The size and shape of the prepared Fe_3_O_4_ and Fe_3_O_4_@SiO_2_ nanoparticles are shown in the TEM images (Figure 3). Their surface area was measured using the BET method, as shown in Table 2. The size of the prepared Fe_3_O_4_ was approximately 237.9 nm, and its shape appeared to be almost spherical with a rough surface. Its surface area was measured to be 21.57 m^2^/g. In the case of the Fe_3_O_4_@SiO_2_ nanoparticles, an SiO_2_ layer (size range = 217.7–236.8 nm) was observed on the surface of the Fe_3_O_4_ core. This is the result of the condensation of hydrolyzed silane precursor (Si(OH)_4_) with itself and the activated site (Fe-OH) on the surface of the Fe_3_O_4_ core. The thickness of the SiO_2_ layer of the dense Fe_3_O_4_@SiO_2_ was the thinnest (24.7 nm), and those of the porous and dual Fe_3_O_4_@SiO_2_ were similar (66.8 nm for porous and ~52.1–60.0 nm for dual) to each other. Additionally, the SiO_2_ shell of the dual Fe_3_O_4_@SiO_2_ comprised a dual layer with a thickness of ~15.6–27.9 nm, and ~24.2–44.4 nm for the dense and porous layers, respectively. Almost spherical shapes were observed for the porous Fe_3_O_4_@SiO_2_ and dual Fe_3_O_4_@SiO_2_, and a slightly rougher structure was observed for the dense Fe_3_O_4_@SiO_2_. The surface areas were measured to be 24.86, 34.50, and 29.58 m^2^/g for the dense, porous, and dual Fe_3_O_4_@SiO_2_ nanoparticles, respectively. The TEM images show that the CTAB was successfully used to build a porous-structured SiO_2_ layer, which resulted in a thicker SiO_2_ layer and a larger surface area with nanosized pores [12]. The SiO_2_ shell of the porous Fe_3_O_4_@SiO_2_ was thicker than that of the porous layer of the dual Fe_3_O_4_@SiO_2_ and similar to the total thickness of the SiO_2_ shell. The dual Fe_3_O_4_@SiO_2_ had two structures for the SiO_2_ coating layer; therefore, it exhibited the thickest SiO_2_ layer. However, during the SiO_2_ fabrication, two types of reaction were conducted: one formed an SiO_2_ layer from the SiO_2_ precursor using the Stöber method based on the sol–gel reaction, and the other layer was fabricated by etching the SiO_2_ layer into the SiO_2_ precursor with an ammonia solution [28]. Therefore, the total thickness of the SiO_2_ layers on porous Fe_3_O_4_@SiO_2_ and dual Fe_3_O_4_@SiO_2_ was observed to be similar owing to a dynamic equilibrium between the SiO_2_ precursor and the SiO_2_ layer.

**Figure 4:**
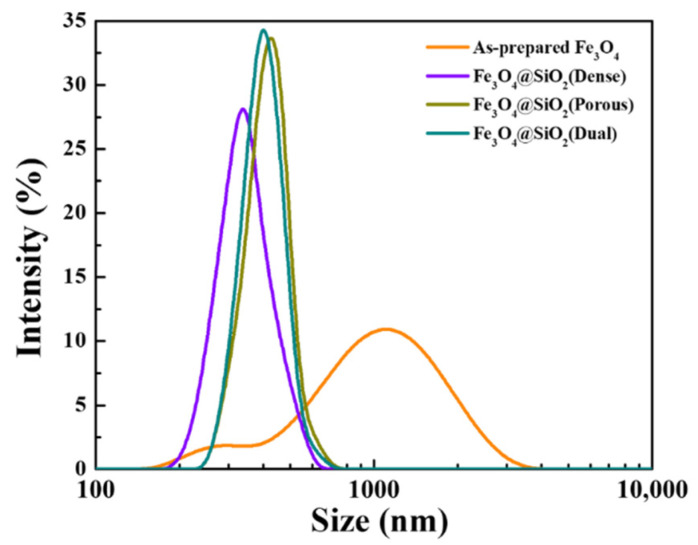
Particle size distribution of as-prepared Fe_3_O_4_ and Fe_3_O_4_@SiO_2_ nanoparticles.

The particle size distributions of the prepared Fe_3_O_4_ and Fe_3_O_4_@SiO_2_ nanoparticles were analyzed using the DLS method and are shown in Figure 4. In the case of the prepared Fe_3_O_4_ nanoparticles, a bimodal parabola was observed with its first peak at 295 nm and second peak at 1106 nm, and it is implied that the particles aggregated along this bimodal parabola. However, all the parabolas of the Fe_3_O_4_@SiO_2_ nanoparticles achieved a monomodal form, and the center axes of these parabolas were 338, 424, and 402 nm for the dense, porous, and dual Fe_3_O_4_@SiO_2_, respectively. The mean size of the prepared nanoparticles was measured to be 1122 nm, which was much larger than that of the actual particle size as observed from the TEM image. Assuming the second peak of size distribution to be due to aggregated particles, the size of the prepared Fe_3_O_4_ was determined to be ~295 nm, which was the center axis of the first peak. This was similar to 238 nm, which was the observed particle size on the TEM image. Assuming that only the SiO_2_ layer was affected by the particle size, the increment in the mean size was double the timed shell thickness when the particle size distribution was compared with that of the TEM images. The increments in the mean sizes were 49, 121, and 107 nm for the dense, porous, and dual Fe_3_O_4_@SiO_2_ nanoparticles, respectively. These were adequate for the thicknesses of the SiO_2_ layers observed on the TEM images. Furthermore, at the point of dispersion, the parabolas of the porous Fe_3_O_4_@SiO_2_ and dual Fe_3_O_4_@SiO_2_ were observed to be very similar, but these parabolas were narrower than those of the dense Fe_3_O_4_@SiO_2_. To be more precise, the polydispersity indexes (PDI) were measured to be 0.617 for the prepared Fe_3_O_4_, 0.204 for dense, 0.159 for porous, and 0.152 for dual Fe_3_O_4_@SiO_2_. Therefore, it was confirmed that Fe_3_O_4_@SiO_2_ had better dispersion properties than those of the prepared Fe_3_O_4_ because a lower PDI results in better dispersion of the particles. The biggest difference in these dispersion properties was the aggregation of the prepared Fe_3_O_4_ nanoparticles that resulted from the lack of repulsive force because of the neutral state of the surface charge. Contrarily, in the case of the Fe_3_O_4_@SiO_2_ nanoparticles, a strong electrostatic repulsive force was provided by the coated SiO_2_, which is one of the most representative materials with a negative surface charge. The zeta potential was measured as −51.2 mV for the dense Fe_3_O_4_@SiO_2_, and −49.1 mV and −50.8 mV for the porous and dual Fe_3_O_4_@SiO_2_, respectively. Therefore, these nanoparticles had a stable dispersion solution with a strong repulsive force. To compare the Fe_3_O_4_@SiO_2_ nanoparticles, some factors (including the structure of the nanoparticles and the amount of remaining trapped surfactant) that affected their dispersion properties were determined. With respect to the structure of the nanoparticles, the nanopores on the SiO_2_ shell affected their dispersion properties by increasing the surface area of the particles. This increased surface area led to an increase in the repulsive force for particle dispersion, so the particles agglomerated easily. In terms of the repulsive force, the amount of trapped surfactant enhanced the steric force via a carbon chain, which was connected to the surface of the nanoparticles. However, these factors did not have a significant impact on the dispersion properties because the effect of the electrostatic repulsive force was much stronger than the effects of the structure of the nanoparticles and the amount of trapped surfactant.

**Figure 5:**
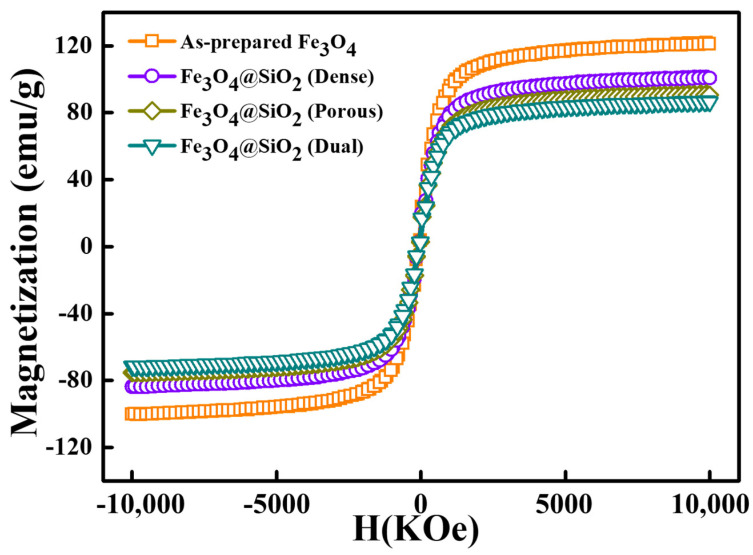
Hysteresis loops of as-prepared Fe_3_O_4_ and Fe_3_O_4_@SiO_2_ nanoparticles.

The magnetic properties of the prepared Fe_3_O_4_ and Fe_3_O_4_@SiO_2_ nanoparticles were determined as hysteresis loops at room temperature, as shown in Figure 5. The hysteresis loops exhibited paramagnetic properties with low remanent magnetization and coercivity [29]. However, unlike other paramagnetic materials, saturated magnetization of each hysteresis loop was observed to be ~80 emu/g, which was high enough for these nanoparticles to exhibit paramagnetic behavior. The saturated magnetization of the prepared Fe_3_O_4_ nanoparticles was 121.7 emu/g (strongest magnetization), and it changed to 101.3, 91.3, and 86.9 emu/g for the dense, porous, and dual Fe_3_O_4_@SiO_2_, respectively. The reduction in the saturated magnetization of the dense Fe_3_O_4_@SiO_2_ was smaller than that of the porous and dual Fe_3_O_4_@SiO_2_, which resulted from the thinner SiO_2_ layer on the dense Fe_3_O_4_@SiO_2_. However, the porous Fe_3_O_4_@SiO_2_ exhibited a higher saturated magnetization than that of the dual Fe_3_O_4_@SiO_2_, which had a thinner SiO_2_ shell. The SiO_2_ shell of the dual Fe_3_O_4_@SiO_2_ had a higher density, which resulted in a lower proportionate weight of the Fe_3_O_4_ core in the dual Fe_3_O_4_@SiO_2_ compared with that of the porous Fe_3_O_4_@SiO_2_. These results indicate that the reduction in the saturated magnetization of the Fe_3_O_4_@SiO_2_ nanoparticles was derived from the weight proportion of these magnetic nanoparticles. Therefore, the saturated magnetization was reduced with the increasing thickness of the SiO_2_ coating layer. However, their saturated magnetization was still high enough and the superparamagnetic properties were maintained, which were essential factors for their application.

**Figure 6:**
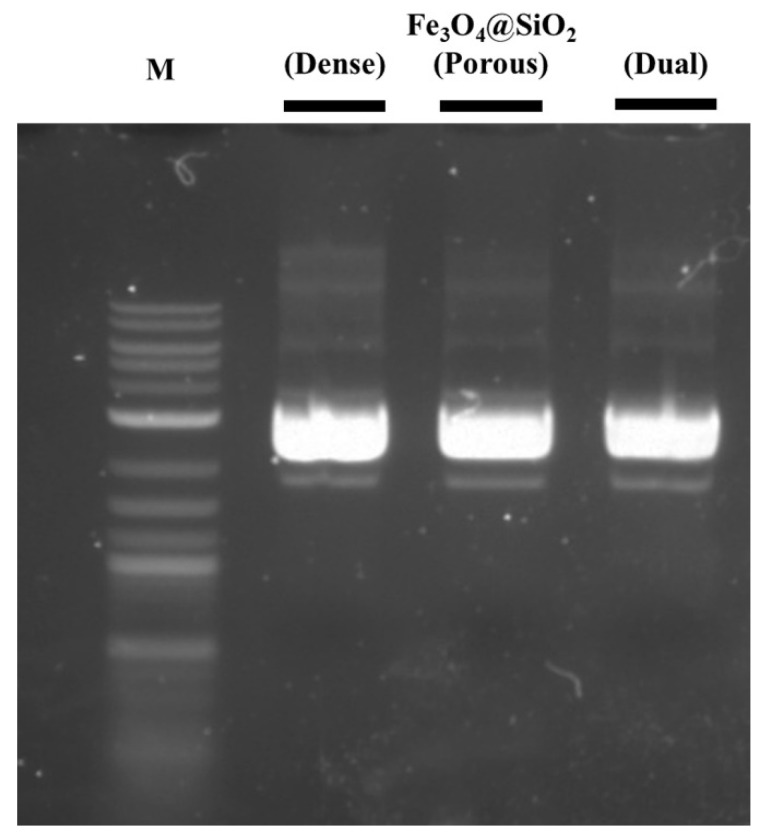
Agarose gel electrophoresis of separated plasmid DNA with Fe_3_O_4_@SiO_2_ nanoparticles.

The plasmid DNA separation process was carried out based on the Fe_3_O_4_@SiO_2_ nanoparticles, and the DNA was separated using agarose gel electrophoresis to evaluate the efficiency of the plasmid DNA purification (Figure 6.). The purity of the plasmid DNA extracted with every Fe_3_O_4_@SiO_2_ nanoparticle was sufficiently high for commercial applications (A260/A280 = 1.985 (dense), 1.924 (porous), and 1.952 (dual)). However, the amount of the separated plasmid DNA differed with the structure of the surface layer. The amounts of DNA separated using the dense Fe_3_O_4_@SiO_2_, porous Fe_3_O_4_@SiO_2_, and dual Fe_3_O_4_@SiO_2_ were measured to be 52.3 ± 10.8 ng/μL, 58.3 ± 5.2 ng/μL, and 61.2 ± 2.4 ng/μL, respectively. The amount of separated DNA increased with the changes in the shell structure of the Fe_3_O_4_@SiO_2_ nanoparticles (into porous and dual-layer structures). According to the surface area of the Fe_3_O_4_@SiO_2_ nanoparticles and the amount of the separated DNA, the DNA separation capability of the Fe_3_O_4_@SiO_2_ nanoparticles seems to be related to the surface area, which was at a maximum for the porous SiO_2_ fabrication. The surface area of the porous Fe_3_O_4_@SiO_2_ was larger than that of the dual Fe_3_O_4_@SiO_2_, and the separated amount of plasmid DNA was smaller than that of the dual Fe_3_O_4_@SiO_2_. Comparing with the surface area and the amount of separated DNA of the dense Fe_3_O_4_@SiO_2_, the surface areas of the porous Fe_3_O_4_@SiO_2_ and dual Fe_3_O_4_@SiO_2_ increased to 138.7% and 119.0%, respectively, whereas the amounts of separated DNA were 111.7% and 117.0%, respectively. In this comparison, increased surface area was not fully related to the DNA separation properties. This difference occurred because the surface areas, which were measured using the BET method, contained inaccessible areas for the plasmid DNA. In the case of the dual Fe_3_O_4_@SiO_2_, this inaccessible area for DNA was smaller than that of the porous dual Fe_3_O_4_@SiO_2_. Although the increased surface area was not fully utilized for DNA separation, plasmid DNA separation capabilities were enhanced in the porous and dual Fe_3_O_4_@SiO_2_. Furthermore, considering the point of efficiency between surface area and DNA separation capability, the dual Fe_3_O_4_@SiO_2_ showed the most reasonable DNA separation property.

## 4. Conclusions

This study proposed a fabrication method for complex-structured Fe_3_O_4_@SiO_2_ nanoparticles while controlling the density of the SiO_2_ shell. The resulting complexes were compared with respect to various properties. To build a dual-layered SiO_2_ shell with different densities, two types of SiO_2_ fabrication method were utilized on the surface of the Fe_3_O_4_ nanoparticles, and these types of coatings were varied by applying CTAB as a temporary structure. After fabrication, differently structured SiO_2_ coating layers were successfully formed on the surface of the Fe_3_O_4_ nanoparticles. In the case of the dual Fe_3_O_4_@SiO_2_, the thicknesses of the SiO_2_ shell were observed to be ~15.6–27.9 nm for the dense layer and ~24.2–44.4 nm for the porous layer. The dual-layered Fe_3_O_4_@SiO_2_ nanoparticles had good dispersion properties with a PDI value of 0.167, and their superparamagnetic properties with high saturated magnetization were also measured (86.9 emu/g). The surface area of the porous Fe_3_O_4_@SiO_2_ was observed to be the largest at 34.50 m^2^/g, which was 16% larger than that of the dual Fe_3_O_4_@SiO_2_ (29.58 m^2^/g). Nevertheless, the DNA separation capability of the dual Fe_3_O_4_@SiO_2_ was measured as 61.2 ng/μL which was a 4% higher amount than that of the porous Fe_3_O_4_@SiO_2_ without affecting the purity of the separated DNA. With this result, a possibility has been identified for applying the dual Fe_3_O_4_@SiO_2_, which exhibited maximum plasmid DNA separation ability with a higher surface area efficiency for the separation of biomaterials. However, since the stability of this nanocomposite has not been proven in this paper, there is also the necessity for further study into the stability of this nanocomposite.

## Figures and Tables

**Table 1:** The FT-IR peak assignment for as-prepared Fe_3_O_4_ and Fe_3_O_4_@SiO_2_ nanoparticles between 500 and 2000 cm.

Types of Bond	Wavenumber
Fe-O bond	600 cm^−1^
C-C bond	808 cm^−1^
C-H bond	880 cm^−1^ 960 cm^−1^ 1470 cm^−1^
Si-O-Si bond	1070 cm^−1^ 1210 cm^−1^
N-H bond	1630 cm^−1^

**Table 2:** The size of the Fe_3_O_4_ core and SiO_2_ shell, and surface area of the as-prepared Fe_3_O_4_ and Fe_3_O_4_@SiO_2_ nanoparticles.

	As-prepared Fe_3_O_4_	Fe_3_O_4_@SiO_2_ (Dense)	Fe_3_O_4_@SiO_2_ (Porous)	Fe_3_O_4_@SiO_2_ (Dual)
Core size	237.9 nm	217.7 nm	236.8 nm	218.7 nm
Shell size	-	24.7 nm	66.8 nm	15.6~27.9 nm(Dense) 24.2~44.4 nm(Porous)
Surface area	21.57 m^2^/g	24.86 m^2^/g	34.50 m^2^/g	29.58 m^2^/g
